# Supporting Patients Treated for Prostate Cancer: A Video Vignette Study With an Email-Based Educational Program in General Practice

**DOI:** 10.2196/jmir.3003

**Published:** 2014-02-26

**Authors:** Moyez Jiwa, Georgia Halkett, Xingqiong Meng, Vinita Pillai, Melissa Berg, Tim Shaw

**Affiliations:** ^1^Curtin UniversityPerthAustralia; ^2^University of SydneySydneyAustralia

**Keywords:** medical education, prostate cancer, general practice, email, video

## Abstract

**Background:**

Men who have been treated for prostate cancer in Australia can consult their general practitioner (GP) for advice about symptoms or side effects at any time following treatment. However, there is no evidence that such men are consistently advised by GPs and patients experience substantial unmet need for reassurance and advice.

**Objective:**

The intent of the study was to evaluate a brief, email-based educational program for GPs to manage standardized patients presenting with symptoms or side effects months or years after prostate cancer treatment.

**Methods:**

GPs viewed six pairs of video vignettes of actor-patients depicting men who had been treated for prostate cancer. The actor-patients presented problems that were attributable to the treatment of cancer. In Phase 1, GPs indicated their diagnosis and stated if they would prescribe, refer, or order tests based on that diagnosis. These responses were compared to the management decisions for those vignettes as recommended by a team of experts in prostate cancer. After Phase 1, all the GPs were invited to participate in an email-based education program (Spaced Education) focused on prostate cancer. Participants received feedback and could compare their progress and their performance with other participants in the study. In Phase 2, all GPs, regardless of whether they had completed the program, were invited to view another set of six video vignettes with men presenting similar problems to Phase 1. They again offered a diagnosis and stated if they would prescribe, refer, or order tests based on that diagnosis.

**Results:**

In total, 64 general practitioners participated in the project, 57 GPs participated in Phase 1, and 45 in Phase 2. The Phase 1 education program was completed by 38 of the 57 (59%) participants. There were no significant differences in demographics between those who completed the program and those who did not. Factors determining whether management of cases was consistent with expert opinion were number of sessions worked per week (OR 0.78, 95% CI 0.67-0.90), site of clinical practice (remote practice, OR 2.25, 95% CI 1.01-5.03), number of patients seen per week (150 patients or more per week, OR 10.66, 95% CI 3.40-33.48), and type of case viewed. Completion of the Spaced Education did impact whether patient management was consistent with expert opinion (not completed, OR 0.88, 95% CI 0.5-1.56).

**Conclusions:**

The management of standardized patients by GPs was particularly unlikely to be consistent with expert opinion in the management of impotence and bony metastasis. There was no evidence from this standardized patient study that Spaced Education had an impact on the management of patients in this context. However, the program was not completed by all participants. Practitioners with a greater clinical load were more likely to manage cases as per expert opinion.

## Introduction

Prostate cancer has been the most commonly diagnosed cancer in Australian men since 1989 [1]. One in nine men in Australia will develop prostate cancer in their lifetime [2]. Most men with prostate cancer survive more than 5 years and die of unrelated causes [3]. The treatment of prostate cancer may include surgery, radiotherapy, chemotherapy, hormone treatment, or watchful waiting. Treatment depends on prognosis, stage of disease, treatment options, and side effects as well as the patient and his partner’s preferences [4].

In the months and years following treatment, men may experience a number of troublesome side effects, or in the case of advanced disease, symptoms and signs related to metastatic disease. These include impotence, urinary incontinence, proctitis, depression, fatigue, and malignant bone pain [5]. Post-treatment follow-up is provided in the tertiary settings in some instances; however, this follow-up may only be for a short period of time after which patients are encouraged to see their general practitioner (GP) about any ongoing problems. Previous studies have demonstrated that men consult a GP routinely in the months and years after treatment for prostate cancer [6]. Prostate cancer patients are more likely to contact their GP for urinary problems and erectile dysfunction (ED) than for other symptoms [6]. GP presentation for fatigue is also more common in prostate cancer patients [6]. However, there is no evidence that such patients are appropriately advised by general practitioners, and patients experience substantial unmet need for reassurance and advice [7]. In order to address the needs of patients treated for prostate cancer, the general practitioner needs to be knowledgeable about the recommended treatment for side effects of radiation therapy and the signs and symptoms that merit urgent referral for further specialist treatment. There is some evidence that general practitioners require further education on the specific needs of men living with prostate cancer and especially those who have received radiation therapy [8,9].

## Methods

### Participants

Following approval from the Curtin Human Research Ethics Committee (HR 08/2011), participants were recruited from a network of 150 GPs across Australia. GPs were emailed invitations and the initial form emails were supplemented with follow-up personal invitations to some of the 150 invitees who did not initially respond. Participants were remunerated with AUD $300 for their contribution.

### Materials

**Figure 1:**
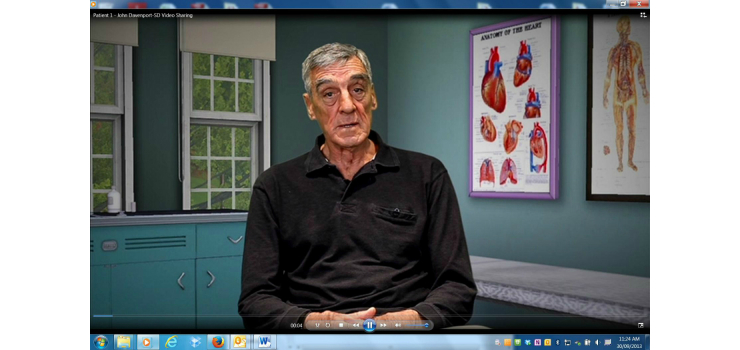
Screenshot of video vignette.

Twelve video vignettes were developed, one pair for each potential side effect related to treatment for prostate cancer or the features of metastasis (see Multimedia Appendices 1 and 2 for exemplars). Each vignette depicted a patient with clear indications for specific management, including referral, prescription, reassurance, and/or investigation. The vignettes were developed by three GPs, a radiation oncologist, a medical oncologist, and a urologist. The expert panel also suggested the management of each case with details of prescription, referral for specialist treatment, and laboratory investigation (Multimedia Appendix 3). The vignettes were then prepared as a short video monologue by an actor-patient (Figure 1). The video included an off-camera commentary by an actor-doctor describing relevant signs to be found on clinical examination. Participation in the study was via the Internet. Participants were asked 4 questions after watching each video vignette: (1) “What is your diagnosis?”, (2) “Would you prescribe something? If so, what?”, (3) “Would you refer the patient? If so, to whom?”, and (4) “Would you order tests? If so, which tests?”

### Development of Spaced Education (Spaced Ed) Program

The program involves clinicians answering small numbers of case-based multiple choice questions that are emailed to them over a number of days. Participants receive one question at a time. Reponses can be submitted in one of two ways either by selecting from a choice of potential answers or by entering a 140 character free-text reply. The questions can be received by email or via a smartphone. The questions are repeated using an evidence-based adaptive algorithm that personalizes the spacing and content of a course to the demonstrated knowledge level of each learner. Learners receive succinct feedback after answering each case and can see how they are progressing through a program and compare their performance with peers. A program is completed when a participant answers all questions correctly twice consecutively. Feedback to the participants included references to the literature where the participants could read more about the subject if they wished. Completing a Spaced Education (Ed) program requires a few minutes every other day to answer the questions. Through the use of this technology, large numbers of geographically distributed practitioners can be reached relatively easily. Spaced Ed has previously been found in many studies to have an impact on knowledge and clinician behavior. A large randomized study investigating the impact of Spaced Ed on inappropriate PSA (prostate-specific antigen) screening by primary care clinicians in the Veterans Affairs Network in the United States found Spaced Ed significantly reduced inappropriate screening and this effect persisted for 2 years after the course [10]. A further randomized study in the United States found Spaced Ed impacted significantly on self-reported global clinical behaviors among primary care clinicians [11]. Spaced Ed is based on the spacing effect—the psychological finding that educational encounters that are spaced and repeated over time result in improved retention and more efficient learning compared to an educational event held at a single point in time [12,13].

The case studies and answers to the questions for the Spaced Ed program deployed in this study were developed by the same multidisciplinary team of clinicians involved in devising the video vignettes. The focus of the case studies matched the materials presented in the video vignettes as described above.

The project was completed in three stages: (1) Phase 1 - participants were invited to view the first set of 6 videos and to describe their management of the standardized patient depicted, (2) Spaced Education - all participants were invited to take part in the Spaced Ed program for 8 weeks, and (3) Phase 2 - all participating GPs were invited to view the second set of six videos and to describe their management of the standardized patient depicted.

### Statistical Analysis

We estimated that approximately 50% of the participants would complete the Spaced Ed program in the specified 8 weeks. The proportion of those who managed cases as per the expert recommendations were expected to be greater in the Spaced Ed group (0.60 vs 0.30). For this reason, a sample of 42 participants per group was deemed sufficient in this exploratory study to estimate the effect size of the Spaced Ed program within 95% confidence intervals ranging from 0-34% [14].

Fisher’s exact tests were used to determine group differences in the proportion of cases diagnosed and managed correctly. Binary logistic regression was used to determine group differences in the correct management of cases by the participants. The full regression model included: the 2 participant groups; age; gender; country of graduation; years after graduation; years of GP experience; status as established GP or GP registrar (trainee); fellowship status with the Royal Australian College of General Practitioners (FRACGP); the remoteness of their primary practice; the number of GPs at their primary practice; status as a principal within their primary practice; patients seen per week; patient care hours per week; and whether they conduct non-English consultations. Regression models were constructed using both backwards elimination and forward selection. Variables with a *P* value less than .05 were retained in the final model and reported, with the exception of the variable of the intervention group, which remained in the model regardless of its significance level. Stata version 12.1 (StataCorp LP, College Station, TX, USA) was used to perform the analysis. Logistic regression models were adjusted for the lack of independence between individual participants by estimating the clustered standard errors to account for intra-group correlation (“vce” option in Stata).

## Results

In total, 64 general practitioners consented to participate in the project, 57 GPs completed Phase 1, 45 completed Phase 2, and 38 of 57 (59%) participants completed the Spaced Education program. There were no significant differences in demographics between those who completed the Spaced Ed program and those who did not (Table 1). There were significant differences in the correct diagnosis of individual cases between the study’s two phases (Table 2). There were some statistically significant differences in the management of cases in Phase 1 compared to Phase 2 (Table 3). In Phase 2, there was no difference in the diagnosis of cases regardless of whether the participant had completed Spaced Ed or not (Table 4). Similarly, there was no difference in the management of cases, whether the participant had completed the Spaced Ed or not (Table 5).

Regression analysis was carried out to determine whether the GPs managed the case as recommended by experts with reference to three explanatory variables: (1) GP demographics, (2) Spaced Education, and (3) Cases. These variables explained 25% of the differences observed (*R* ^*2*^=.25) (Table 6). The number of sessions in general practice was strongly correlated with the number of patients seen per week (Pearson’s correlation coefficient of .74, *P*<.001). However, the number of sessions in practice were inversely correlated with correct case management. It is possible that some of the practitioners who were seeing a greater number of patients had received specialist training in prostate cancer; however, we could not test this hypothesis from these data. Male GPs did more clinical sessions (median 8, IQR 6) than females (median 6, IQR 6); however, gender did not have a significant influence in the regression analysis.

**Table 1:** Participant demographic information.

Characteristics			Total sample (n=64)	Spaced Ed, completed (n=38)	Spaced Ed, not completed (n=26)	*P* value
			mean (SD) or n (%)	mean (SD) or n (%)	mean (SD) or n (%)	
**Participant demographics**						
		Age (years)	43.5 (11.3)	41.8 (10.7)	46.1 (11.9)	.14
		Years after graduation	19.8 (11.5)	-	-	.11
		Years of GP experience	15.0 (11.8)	13.2 (11.2)	17.5 (12.5)	.16
		Number of other GPs at same clinic	7.4 (4.2)	6.6 (3.5)	8.5 (4.9)	.07
		GP sessions worked/week	6.8 (2.8)	7.1 (2.7)	6.4 (3.0)	.36
		Male	34 (53.1%)	18 (47.4%)	16 (61.5%)	.31
		Graduated in Australia	48 (75.0%)	27 (71.1%)	21 (80.8%)	.56
		Registrars	14 (21.9%)	7 (18.4%)	7 (26.9%)	.54
		FRACGP^b^	34 (53.1%)	21 (55.3%)	13 (50.0%)	.80
**Primary practice demographics**						
	Accredited		63 (98.4%)	38 (100.0%)	25 (96.2%)	.41
	**Location**					.35^a^
		ACT (Australian Capital Territory)	1 (1.6%)	1 (2.6%)	0 (0.0%)	
		NSW (New South Wales)	10 (15.6%)	8 (21.1%)	2 (7.7%)	
		QLD (Queensland)	5 (7.8%)	3 (7.9%)	2 (7.7%)	
		SA (South Australia)	5 (7.8%)	3 (7.9%)	2 (7.7%)	
		TAS (Tasmania)	1 (1.6%)	0 (0.0%)	1 (3.9%)	
		VIC (Victoria)	14 (21.9%)	10 (26.3%)	4 (15.4%)	
		WA (Western Australia)	26 (43.8%)	13 (34.2%)	15 (57.7%)	
	**Clinic remoteness**					.93
		Major city	44 (68.8%)	26 (68.4%)	18 (69.2%)	
		Inner regional	9 (14.1%)	6 (15.8%)	3 (11.5%)	
		Outer regional/remote	11 (17.2%)	6 (15.8%)	5 (19.2%)	
	**GP position**					.13
		Principal	15 (23.4%)	6 (15.8%)	9 (34.6%)	
		Non-Principal	39 (60.9%)	27 (71.1%)	12 (46.1%)	
		Others	10 (15.6%)	5 (13.2%)	5 (19.2%)	
**Patient consultations**						
	**Patient consultations per week**					.80
		<100	32 (50.0%)	18 (47.4%)	14 (53.9%)	
		100-149	18 (28.2%)	12 (31.6%)	6 (23.1%)	
		≥150	14 (21.9%)	8 (21.1%)	6 (23.1%)	
	**Patient consultation hours per week**					1.00
		<11	7 (10.9%)	4 (10.5%)	3 (11.5%)	
		11-20	11 (17.2%)	7 (18.4%)	4 (15.4%)	
		21-40	35 (54.7%)	21 (55.3%)	14 (53.9%)	
		≥41	11 (17.2%)	6 (15.8%)	5 (19.2%)	
	**Non-English consultations**					.53
		No	52 (81.3%)	32 (84.2%)	20 (76.9%)	
		<25%	12 (18.8%)	6 (15.8%)	6 (23.1%)	

^a^ *P* values were derived from Fisher’s exact test.

^b^FRACGP: Fellowship Royal Australian College of General Practitioners

**Table 2:** Correct diagnosis of cases per phase of study.

Diagnosis		Phase 1, correct (n=57), n (%)	Phase 2, correct (n=45), n (%)	*P* value
**Case 1**				
	Radiation proctitis	42 (73.7%)	45 (100.0%)	<.001
**Case 2**				
	PSA bounce after radiation therapy	21 (36.8%)	26 (57.8%)	.04
**Case 3**				
	Spinal metastasis	50 (89.3%)	40 (88.9%)	.86
**Case 4**				
	Urethral stricture after radiotherapy	24 (43.6%)	38 (84.4%)	<.001
**Case 5**				
	Psychological cause	26 (47.3%)	23 (51.1%)	.58
	Adverse effect of medication	10 (18.2%)	19 (42.2%)	.006
**Case 6**				
	Biological depression	39 (70.9%)	10 (22.2%)	<.001
	Psychosocial factors	53 (96.4%)	45 (100.0%)	.07^a^

^a^ *P* values were derived from Fisher’s exact test.

**Table 3:** Correct management of cases by phase of study.

Management		Phase 1, correct (n=57), n (%)	Phase 2, correct (n=45), n (%)	*P* value
**Case 1 (Proctitis)**				
	Refer to specialist	15 (26.3%)	19 (42.2%)	.09
	Rule out bowel infection	17(29.8%)	21 (46.7%)	.08
	Refer for colonoscopy	30 (52.6%)	20 (44.4%)	.41
	Prescribe medication	1 (1.8%)	16 (35.6%)	<.001^a^
**Case 2 (Anxiety)**				
	No specific treatment	20 (35.1%)	17 (37.8%)	.77
	Reassure	4 (7.0%)	1 (2.2%)	.26^a^
**Case 3 (Recurrence)**				
	Refer to radiation oncologist	15 (26.8%)	17 (37.8%)	.22
	Seek specialist advice on investigations	36 (64.3%)	25 (55.6%)	.44
	Order plain x-rays	42 (75.0%)	32 (71.1%)	.77
**Case 4 (Stricture)**				
	Refer to urologist	19 (34.6%)	21 (46.7%)	.17
	Refer to physiotherapist	0 (0.0%)	1 (2.2%)	.25^a^
	Micturating cysto-urethrogram	27 (49.1%)	26 (57.8%)	.29
	Renal ultrasound scan	24 (43.6%)	15 (33.3%)	.36
**Case 5 (Impotence)**				
	Check cholesterol	13 (23.6%)	24 (53.3%)	.001
	Check blood glucose	14 (25.5%)	21 (46.7%)	.02
	Check hormone levels	12 (21.8%)	22 (48.9%)	.006
**Case 6 (Depression)**				
	Prescribe antidepressant	39 (73.6%)	40 (88.9%)	.01

^a^ *P* values derived from Fisher’s exact test.

**Table 4:** Correct diagnosis of cases as per completion of Spaced Ed (SE) in Phase 2 (n=45).

Phase 2		SE completed, n=37		SE not completed, n=8		*P* value^a^
		Diagnosed correctly	Diagnosed incorrectly	Diagnosed correctly	Diagnosed incorrectly	
**Case 1**						
	Radiation Proctitis	100.0	-	100.0	-	-
**Case 2**						
	PSA Bounce after radiation therapy	22	15	4	4	.70
**Case 3**						
	Spinal metastasis	33	4	7	1	1.00
**Case 4**						
	Urethral stricture after radiotherapy	32	5	6	2	.59
**Case 5**						
	Psychological cause	19	18	4	4	1.00
	Adverse effect of medication	15	22	4	4	.70
**Case 6**						
	Biological depression	10	27	0	8	.17
	Psychosocial factors	37	-	8	-	-

^a^All *P* values in this table were derived from Fisher’s exact test.

**Table 5:** Correct management of cases as per completion of Spaced Ed (SE) in Phase 2 (n=45).

Phase 2		SE completed, n=37		SE not completed, n=8		
		Managed as recommended	Not managed as recommended	Managed as recommended	Not managed as recommended	*P* value^a^
**Case 1 (Radiation)**						
	Refer to specialist	17	20	2	6	.43
	Rule out bowel infection	18	19	3	5	.71
	Refer for colonoscopy	16	21	4	4	1.0
	Prescribe medication	-	-	-	-	-
**Case 2 (PSA Bounce)**						
	No specific treatment	13	24	4	4	.45
	Reassure	1	36	0	8	1.0
**Case 3 (Recurrence)**						
	Refer to radiation oncologist	16	21	1	7	.13
	Seek specialist advice on investigations	23	14	2	6	.11
	Order plain x-rays	26	11	6	2	1.0
**Case 4 (Stricture)**						
	Refer to urologist	18	19	3	5	.71
	Refer to physiotherapist	1	36	0	8	1.0
	Micturating cysto-urethrogram	23	14	3	5	.25
	Ultrasound scan	14	23	1	7	.24
**Case 5 (Impotence)**						
	Check cholesterol	21	16	3	5	.44
	Check blood glucose	17	20	4	4	1.0
	Check hormone levels	19	18	3	5	.7
**Case 6 (Depression)**						
	Prescribe antidepressant	32	5	8	8	.27

^a^All *P* values derived from Fisher’s exact test other than cases where cells contained 5 or more participants where Pearson’s chi-square test was used.

**Table 6:** Regression analysis.

Variable		OR	95% CI	*P* value
Sessions worked per week		0.78	0.67-0.90	.001
**GP registrar**				
	Yes	1.00		
	No	4.66	2.23-9.71	<.001
**Clinical remoteness**				
	Major cities	1.00		
	Inner regional	0.38	0.21-0.70	.002
	Outer regional/remote	2.25	1.01-5.03	.048
**Patients seen per week**				
	<100	1.00		
	100-149	4.53	1.91-10.72	.001
	≥150	10.66	3.40-33.48	<.001
**Spaced Education**				
	Completed	1.00		
	Not completed	0.88	0.50-1.56	.66
**Cases**				
	1. Proctitis	1.00		
	2. PSA bounce	5.36	1.79-16.09	.003
	3. Bony metastasis	1.27	0.42-3.82	.67
	4. Urethral stricture	39.75	10.42-151.57	<.001
	5. Impotence	0.67	0.24-1.88	.44
	6. Depression	2.92	1.05-8.15	.04

## Discussion

### Principal Findings

Bowel, urinary or sexual dysfunction, depression, and anxiety are common presentations in primary care (1). In this study, patients with such problems were presented in the context of treatment for prostate cancer. Our data indicate that there were limited numbers of participants who correctly diagnosed the symptoms presented or suggested a management plan that was consistent with expert opinion. In Phase 1, the differences were marked for most cases (Tables 2 and 3). Such deviations from expert opinion have been reported previously [15,16]. From the regression analysis, we were able to conclude that compared to radiation proctitis, PSA bounce, urethral stricture, and depression were more likely to be managed as per the experts. However, erectile dysfunction was less likely to be managed as per expert opinion, especially in Phase 1. Erectile dysfunction is the most common side effect of treatment for early prostate cancer. It has far-reaching effects upon men’s lives [17]. Although some treatment effects such as radiation proctitis are relatively uncommon, ED is a common symptom that is likely to be presented to general practitioners in many clinical contexts [18].

Overall, the differences in management between the participants and the expert panel were less marked in Phase 2 of the study, and it is possible that in the intervening 8 weeks participants may have sought information on how to manage the adverse effects of prostate cancer treatment. The only exception was the diagnosis of biological depression, which seemed to deviate more from expert opinion in Phase 2. This was unexpected and it may have reflected a reticence to diagnose significant depression in that vignette and or it may be that the actor did not display the features of a significant depression in a way that persuaded more practitioners to come to that diagnosis.

The observation that rural practitioners were more attuned to expert management is consistent with the survey of Australian GPs, where rural GPs were more willing to be involved in providing supportive care to cancer patients than colleagues in metropolitan areas [19]. With respect to the main focus of our study, those who completed the Spaced Ed intervention were not more likely to concur with the expert panel. This is in contrast to other previous reported studies [10,11]. The reason for this is unclear, but may relate to the nature of the conditions being considered or the context in which this intervention was delivered. A primary care consultation is known to be complex, with a focus on the physical, social, and psychological components of any symptom or problem presented. This may not lend itself to an intervention that promotes the application of simple rules [20]. Regression analysis suggests that more influential variables impacting on the outcomes were some of the demographic characteristics of the participants; specifically, a greater clinical load. This was not unexpected for patients treated for prostate cancer because many of these problems are likely to present infrequently, and few doctors will have encountered them previously unless consulting a large number of men.

A number of approaches have been reported in the literature to promote consistent and reliable management of chronic conditions in primary care [21,22]. A few of these have focused specifically on the knowledge of general practitioners [23]. Data from our study suggests that focusing on knowledge alone may not be sufficient. A recent literature review reported that two other factors are also likely to be important in the context of a cancer diagnosis, namely, attitudes and beliefs [24,25]. These issues were not evaluated in this study. For example, we were unable to report the participants’ attitude to the management of patients following treatment and whether they felt this role extended to investigating and treating conditions that may have resulted from specialist treatment [9]. The review of attitudes to this issue among Australian GPs suggests that there is a diversity of opinions on the matter [19]. Nor could we confirm that all participants had access to a radiation oncologist in their clinical practice and/or would have had the option to refer a patient with bony metastasis or radiation proctitis to such an expert. The available evidence suggests that this is not a safe assumption and that management plans would be impacted by the clinicians’ experience in their local context [26]. Finally, we could not identify any practitioners who had any specialist training in prostate cancer. However, all participants were working as general practitioners when they participated in this study and it is reasonable to assume that there were a negligible number with specialist training in a specific cancer.

With respect to the format of education offered here, even though more than half the sample completed the Spaced Ed program, it was disappointing that more did not do so. We did not observe any significant differences in the demographic characteristics of those who completed the Spaced Ed program and those who did not. The program was based on email communication and relied on doctors checking emails on a regular basis. There is limited evidence from the Australian literature that general practitioners routinely deploy digital technology [27]. Second, there was no evidence that participants were satisfied that the answers provided to the emailed scenarios were consistent with what they considered best practice. Informal feedback suggested that the program was unpopular even among those who persevered with it. For example, some participants pointed out that they disagreed with the answers offered by the experts.

### Conclusions

In this standardized patient study, Spaced Ed did not promote management plans that were consistent with expert opinion. While there was a marked improvement in the management of cases in Phase 2 of this study, this may be because participants were stimulated to seek information elsewhere on the management of such cases. Greater clinical load had a more significant and positive impact on the management of patients than the Spaced Ed. Further development of the Spaced Ed may be required before it can be tested again in the context of prostate cancer follow-up in general practice. Perhaps greater involvement of the target group of practitioners in setting the answers in Spaced Ed may be helpful. Alternatively, it is also possible that while Spaced Ed as a short, targeted educational program facilitated by information technology is attractive, it is unlikely to succeed.

## Multimedia Appendix 1

Example of video.

## Multimedia Appendix 2

Typical cases.

## Multimedia Appendix 3

Specific recommendations for management of cases.

## Abbreviations

ED: erectile dysfunction
GP: general practitioner
PSA: prostate-specific antigen test
Spaced Ed: spaced education
