# Supplementary material for: Supporting Patients Treated for Prostate Cancer: A Video Vignette Study With an Email-Based Educational Program in General Practice
Source: J Med Internet Res. 2014 Feb 26;16(2):e63. doi: 10.2196/jmir.3003 (PMC3961707; doi:10.2196/jmir.3003)
Supplement: Supplementary file 2 [file jmir_v16i2e63_app2.pdf]

Multimedia Appendix 2 – Typical Cases

Consultation 1: John Davenport, 78 years old, was diagnosed with localized prostate cancer (24 months ago). Treated with external beam radiation therapy. Suffering from symptoms of faecal urgency and frequency several times a day from past one month with specks of blood and cramping. Feeling anxious due to incontinence in public. On examination found to have painful, tight rectum, fresh blood and stool on gloves. **Radiation proctitis**

Consultation 2: Paul Sutcliffe, 68 years old. Feeling tired and anxious. Diagnosed with localized prostate cancer (12 months ago). Treated with brachytherapy. PSA since treatment: 6 weeks post-treatment: 2ng/m, 7 months post-treatment 1.6ng/ml. Last PSA blood test 1 week ago, result 2.7ng/I. He wants to know if it is cancer recurrence. Examination is normal. **PSA bounce**

Consultation 3: Tom Hayden, 68 years old. Father died of prostate cancer at the age of 51 years. Diagnosed with localised prostate cancer (4 years ago). Treated with hormone treatment (6 months), external beam radiation therapy, and brachytherapy. Recently suffering from pain in upper thigh and swelling on ankles. Feels tired due to sleep disturbance as a result of pain. Last PSA blood test 1 week ago, result: 52ng/I. On examination moderate ankle oedema inguinal lymphadenopathy. **Recurrence with bony metastasis**.

Consultation 4: Henry Kendall, 68 years old retired bookkeeper. History of urinary tract infection treated with antibiotics. Diagnosed with localised prostate cancer (1.5 years ago). Treated with hormone treatment (6 months), external beam radiation therapy and HDR brachytherapy x3. He is suffering from symptoms of frequency several times during day and night, hesitancy, urgency, and incontinence over the past three weeks. He is also finding it difficult to initiate urination. He is not on any medication. On examination no abnormalities found: no discharge, no redness or swelling, no tenderness on examination. No varicocele, but leaked a few drops of clear urine with cough. **Urethral stricture.**

Consultation 5: Christopher Lovell, 72 years old. Diagnosed with localised prostate cancer (6 years ago). Treated with brachytherapy, since he could cope with anaesthetic and he couldn’t make the frequent visits required for external beam radiation therapy. He has trouble getting and maintaining an erection. He is a non-smoker and on no medication. On examination no abnormalities found: no discharge, no redness or swelling, no tenderness with examination. Urethral normal size, penis normal. Testis normal size. **Impotence.**

Consultation 6: Terrance Howard, 72 year old. Feeling listless, tired, doesn’t want to socialize. Complains of insomnia. He is worried, frustrated, and depressed. Dwelling on the idea that the prostate cancer might recur. Minimal social support. On examination no physical abnormalities. **Depression.**
