# Supplementary material for: Supporting Patients Treated for Prostate Cancer: A Video Vignette Study With an Email-Based Educational Program in General Practice
Source: J Med Internet Res. 2014 Feb 26;16(2):e63. doi: 10.2196/jmir.3003 (PMC3961707; doi:10.2196/jmir.3003)
Supplement: Supplementary file 3 [file jmir_v16i2e63_app3.pdf]

Multimedia Appendix 3 - Specific recommendations for management of cases.

Case	Description	Recommended management
1.	Radiation proctitis	*Refer to Specialist*: Refer back to radiation oncologist;<br>*Order Test 1*: Send stool sample for culture and sensitivity;<br>*Order Test 2*: Refer for flexible sigmoidoscopy or colonoscopy.<br>*Prescribe medication:* Prescribe diphenoxylate hydrochloride and atropine sulphate (Lomotil)
2.	PSA bounce	No specific medication or other treatment indicated; reassure patient.
3.	Bony metastasis	*Refer to Specialist 1*: Refer urgently to radiation oncologist;<br>*Refer to Specialist 2*: Seek specialist guidance on further investigations;<br>*Order Test*: plain X-rays.
4.	Urethral Stricture	*Refer to Specialist 1*: Refer to urologist for possible surgical treatment;<br>*Refer to Specialist 2*: Refer to a physiotherapist for pelvic floor exercise;<br>*Order Test 1*: micturating cysto urethrogram (MCU);<br>*Order Test 2*: renal ultrasound scan.
5.	Impotence	*Order Test 1*: cholesterol;<br>*Order Test 2*: blood glucose;<br>*Order Test 3*: testosterone levels.
6.	Depression	*Prescribe medication*: antidepressant
